# Optimized Copper-Based Microfeathers for Glucose Detection

**DOI:** 10.3390/bios13121032

**Published:** 2023-12-15

**Authors:** Carlota Guati, Lucía Gómez-Coma, Marcos Fallanza, Inmaculada Ortiz

**Affiliations:** Chemical and Biomolecular Engineering Department, University of Cantabria, 39005 Santander, Spain; guatic@unican.es (C.G.); gomezcomal@unican.es (L.G.-C.); fallanzam@unican.es (M.F.)

**Keywords:** glucose detection, stability, microfeathers, copper, sensitivity

## Abstract

Diabetes is expected to rise substantially by 2045, prompting extensive research into accessible glucose electrochemical sensors, especially those based on non-enzymatic materials. In this context, advancing the knowledge of stable metal-based compounds as alternatives to non-enzymatic sensors becomes a scientific challenge. Nonetheless, these materials have encountered difficulties in maintaining stable responses under physiological conditions. This work aims to advance knowledge related to the synthesis and characterization of copper-based electrodes for glucose detection. The microelectrode presented here exhibits a wide linear range and a sensitivity of 1009 µA∙cm^−2^∙mM^−1^, overperfoming the results reported in literature so far. This electrode material has also demonstrated outstanding results in terms of reproducibility, repeatability, and stability, thereby meeting ISO 15197:2015 standards. Our study guides future research on next-generation sensors that combine copper with other materials to enhance activity in neutral media.

## 1. Introduction

Since COVID-19, healthcare accessibility and well-being have become important issues worldwide, especially in developing countries [1]. Before the pandemic, the main challenges to improving the health of the global population focused on increasing life expectancy and decreasing maternal and childhood mortality. However, the recent health emergency has highlighted other critical needs related to non-communicable diseases and their consequences [2]. These aspects are considered in the Sustainable Development Goals Agenda, notably in goal number 3, which specifies the control of non-communicable diseases as a target for 2030.

The World Health Organization (WHO) considers diabetes as one of the four major types of non-communicable diseases (cardiovascular disease, diabetes, cancer, and chronic respiratory diseases). The number of people suffering from this pathology has grown over time, from 108 million in 1980 to 422 million in 2014. Apart from the health impact of this disease, it is estimated that people with diagnosed diabetes have medical expenditure approximately 2.3 times higher than expenditure in the absence of diabetes [3]. Thus, it is of vital importance to monitor blood glucose levels for the early diagnosis, prevention, and treatment of the diabetic patient [4].

Nowadays, the glucose sensor field is dominated by electrochemical methods that are represented by invasive and minimally/non-invasive commercial devices. In this context, the electrochemical field for developing glucose sensors has experienced continuous growth throughout the 21st century. In recent years, several reviews have compiled the latest advancements [5,6,7,8,9]. The former is a single-use system which involves several fingers pricks during the day, thereby annoying patients and making it difficult to continuously monitor glucose levels [10]. Minimally and non-invasive devices provide a continuous register of glucose levels, which help patients prevent and control hyperglycemic and hypoglycemic peaks. However, these devices last for only 14 days and must be replaced by new ones [11,12,13]. For example, Heo et al. (2019) provided an insightful comparison of the lifespan of various commercial continuous glucose sensors [13]. These investigators revealed a maximum duration of 14 days and pointed out many sensors with a useful life of 7 days.

Both systems use enzymes to detect glucose in biologically complex fluids and provide great selectivity and sensitivity for this organic molecule. On the other hand, enzyme immobilization procedures are complex, they work under critical operational conditions, and enzymatic systems usually present instability issues that shorten the sensors life [14]. Because of these reasons, minimally/non-invasive electrochemical enzymatic sensors are considered expensive. In this scenario, non-enzymatic nanostructures have gained much attention due to their outstanding properties in mimicking enzyme systems [15]. Several authors have published their results with different non-enzymatic electrodes, which can detect glucose under physiological conditions, such as neutral pH and in the presence of common interferences such as ibuprofen or ascorbic acid [16,17,18]. For example, authors have developed a TiO_2_/PAPBA/Au glucose sensor which measures glucose at neutral pH with good sensitivity [19].

The materials used for non-enzymatic glucose sensors can be common metal structures, complex metallic combinations or even mixtures with carbon derivatives or conductive polymers [20,21,22,23]. Noble metals such as gold and platinum have been comprehensively studied because of their biocompatibility in human biofluids [24], however, high prices and poisoning issues from noble metals have promoted searches for other materials for commercial applications [25]. Today, metal oxides represent reliable alternatives for the sensing field since they are affordable materials with high catalytic capacity and they are easy to manipulate [26]. The application of these materials to multiple fields (environmental, healthcare, or food and beverage industries) has been largely considered because of aforementioned advantages. However, for healthcare, electrodes must be active at neutral pH since all biofluids do not exceed a pH range of 5.5–7.5, and not all metal oxides provide stable responses under these conditions. In this scenario, the scientific community is working intensively to overcome these issues through the implementation of pH control systems in sensors or through combinations with other metals and carbon derivatives. Since glucose sensor technology is moving toward noninvasive skin patches, pH control is a practicable choice for the implementation of metal oxides. These patches control glucose levels by measuring sweat parameters through a capillarity system, where pH control can be implemented [27]. However, the implementation of a pH control system is still under investigation and requires further refinement to minimize additional costs. Several authors have suggested the implementation of NaOH crystals on electrode working areas or the inclusion of a hydroxyl layer adsorbed onto the electrode design without interfering with biofluid characteristics, thus only measured samples will be affected by modifying its pH [28]. Non-enzymatic sensors based on metal oxides could reach the diabetes market in the upcoming future thanks to the effective combination of different catalytic materials or the addition of hydroxyl groups to sensor designs. 

In this work, new results obtained with a copper oxide-based electrode for glucose detection are presented. Copper was selected because of its promising results, abundance, and biocompatibility [29,30], and it also represents a good choice for tumor microenvironments since CuO catalyzes endogenous H_2_O_2_ into O_2_ to relieve tumor hypoxia [31]. This provides copper materials with great potential for medical applications, especially in sensor development based on electrochemistry principles.

Although there is still much work to be done, the outcomes reported here represent a step forward in the application of copper to the non-enzymatic glucose sensor field for its outstanding results and ease of synthesis. 

## 2. Materials and Methods

Materials and methods are reported in this section. The developed synthesis method pays special attention to reproducibility, effectiveness, and environmental aspects.

### 2.1. Apparatus and Chemicals

Copper (II) sulfate pentahydrate (≥99.9%), L-ascorbic acid reagent (99.7%), Ibuprofen (≥98%), Sulfuric acid (≥99.7%), and Nafion TM 117 (5%) were purchased from Sigma-Aldrich Corporation (St. Louis, MO, USA). D(+)-glucose anhydrous (97%), ethanol (absolute pure), potassium chloride, and sodium hydroxide pellets were purchased from PanReac AppliChem. Uric acid (99%) was acquired from Alfa Aesar Chemicals (Haverhill, MA, USA). Hexaammineruthenium (III) chloride (Ru(NH_3_)_6_Cl_3_) was acquired from Sigma Aldrich. All chemicals were utilized without additional purification. Distilled water (18.2 MU cm), purified using the Millipore Advantage A10 water system, was used in all experiments for solution preparation.

### 2.2. Electrode Synthesis and Preparation

The working electrode was prepared by first reconditioning the screen carbon printed electrode (SCPE). It underwent a cleaning process involving washing with distilled water followed immediately by rinsing with pure ethanol. Subsequently, the electrode surface was air-dried under ambient conditions. Prior to the electrodeposition of copper particles, the cleaned surface underwent two cyclic voltammetry runs in the range from −0.6 V to 0.6 V vs. Ag/AgCl at a scan rate of 100 mV s^−1^, utilizing 0.1 M CuSO_4_·5H_2_O and 0.1 M H_2_SO_4_. The solution was carefully poured onto the electrode surface with a micropipette in a volume of 150 µL.

Cu particles were electrodeposited through the chemical reduction of 0.1 M CuSO_4_·5H_2_O in 0.1 M H_2_SO_4_, using a chronoamperometry technique at −0.366 V vs. Ag/AgCl, as per a prior study [32]. The deposition time of Cu particles on the carbon-printed electrode (CPE) was systematically studied using Taguchi methods. It is well known that the electrodeposition time affects electrode performance since a high copper surface thickness may hinder contact between the catalyst and the electrolyte, resulting in reduced responses to glucose [33].

**Figure 1:**
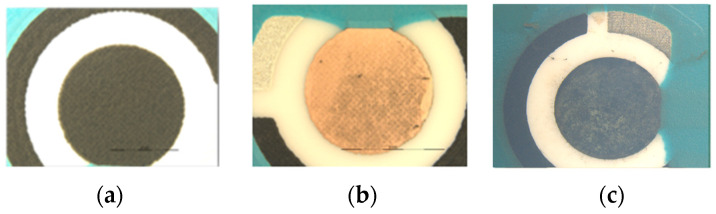
Electrode surfaces. (**a**) SCPE. (**b**) after copper electrodeposition. (**c**) after a 4 day oxidation step.

For the oxidation of copper particles, the electrode was submerged for 4 days in an oxidation medium containing 6 mL of 10 M NaOH, 3 mL 0.2 M (NH_4_)_2_S_2_O_8_, and 21 mL UP water [34]. These variables were optimized following Taguchi methodology, as described in the next section. Figure 1 shows the qualitative color changes of the electrode during the synthesis process. The diameter of the electrode is 2 mm.

Screen carbon printed electrodes with a visual area of 0.12 cm^2^ were purchased from Metrohm Dropsens. All measurements were performed with a Palm-Sens 4 potentiostat galvanostat impedance analyzer. The apparatus had a large potential range (from −10 V to 10 V) and a current range (from 100 pA to 10 mA). PSTrace 7 software was used to collect and display measurements for glucose detection electrodes.

Electrode characterization was conducted using scanning electron microscopy (SEM) and Energy X-ray Powder Diffraction analysis in combination with SEM microscopy EVO MA15 ZEISS^®^ (Germany). SEM images were used to analyze the morphology and structure of the microelectrode surface following the synthesis process. Electrochemical measurements were performed using cyclic voltammetry and pulsed amperometry techniques. For the former, the problem solution was dripped onto the electrode surface and then a specific potential sweep was applied to the working electrode. When applying pulsed amperometry, the electrode was submerged into the problem solution under constant stirring. In this case, a dedicated potential was applied at defined time intervals.

### 2.3. Taguchi Experimental Design

Optimization was used to perfect a designed system; in this sense, several authors have incorporated this aspect to sensor research [35]. The Taguchi method involves reducing the variation of device performance in a process through robust experimental design. The overall objective of this methodology is to produce high quality results, in this case, robust non-enzymatic electrodes. Economy and cost reductions addressed by the Taguchi methodology emphasize the reduction of variations of electrode signals, particularly when total product variation is within the specification limits of the product [36,37]. In this study, the following variables were selected and optimized: CuSO_4_·5H_2_O concentrations, electrode oxidation times, chronoamperometry times, and number of chronoamperometry cycles. Other variables such as the concentration of the oxidative species or the number of cyclic voltammetries before copper electrodeposition were not included because they would complicate the optimization tool. They had less influence on the synthesis process than selected variables, which were directly related to crystal growth synthesis. 

Table 1 presents the selected variables. The selection of these variables was based on their ease of manipulation. Table S1 in Supplementary Materials shows the selected orthogonal array for experiments.

## 3. Results and Discussion

### 3.1. Optimization of the Synthesis Method

This research included methodology optimization to maximize electrode signals in the presence of glucose. Since the synthesis process included several variables, such as precursor concentrations or oxidation times, the determination of exact parameters would be time and resource consuming. By implementing Taguchi tools in research, the scientific community can save time and materials.

**Figure 2:**
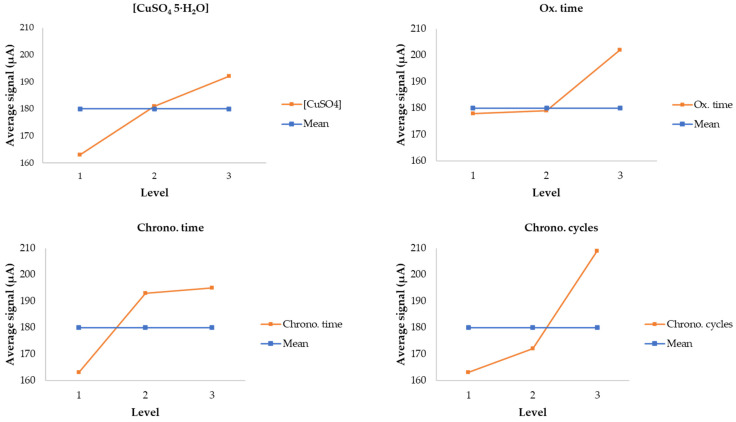
Taguchi study results for the three levels.

Figure 2 depicts the results of the Taguchi analysis using Minitab 20.4 software. The electrochemical technique used was cyclic voltammetry (from −0.6 to 0.6 V vs. Ag/AgCl, 1 cycle) with a solution of 2 mM of glucose in 0.1 M NaOH media. Each electrode was previously conditioned with three cyclic voltammetry runs of 0.1 M NaOH. Each plot represented one studied variable and the mean of all experiments at the selected level (see Table 1). Blue dots represent the mean value of all performed experiments. Thus, the effect of one level could be compared to the average signal. 

As observed, oxidation times and the number of chronoamperometric cycles were the variables that most affected the final electrode signal. Thus, for the remaining experiments, variables were set at 0.1 M CuSO_4_·5H_2_O, three chronoamperometry runs of 300 s, and 4 days of oxidation. These values were chosen considering the trade-off between the maximum signal and minimizing synthesis process times.

### 3.2. Electrode Characterization and Active Area

**Figure 3:**
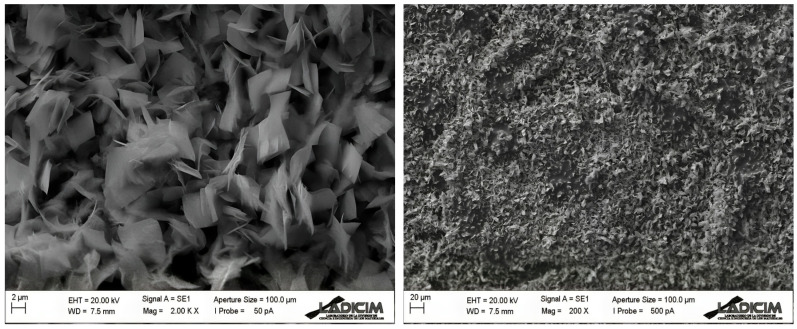
SEM images of the microfeather CuO sensor.

Figure 3 depicts the SEM morphology of synthesized sensors. The image shows the presence of copper oxide microstructures on the carbon surface. The length of copper oxide microfeathers is 1–2 µm. This size is in good agreement with previous results and provides a high increment in electrode surface area [38,39]. The particles were homogeneously dispersed as shown in Figure 3. Figures S1 and S2 in Supplementary Materials show the chemical composition of the studied electrode.

To determine active areas in copper- and copper oxide-based electrodes, Ru(NH_3_)_6_Cl_3_ was selected as an electroactive probe because of its rapid electron transfer capabilities. Several cyclic voltammetry runs were performed varying scan rates from 20 mV/s to 200 mV/s within the range from −0.5 V to 0.8 V to promote the following electrochemical reaction [33]:

[Ru(NH_3_)_6_]^3+^ + e^−^ ↔ [Ru(NH_3_)_6_]^2+^ (1)

Equation (1) for the determination of the active area is applied when the redox process is quasi- or irreversible. For this purpose, a peak to peak distance (ΔEp) analysis of cyclic voltammetry profiles was needed. For those cases where Δep = 57 mV for all scan rates, the redox process was classified as reversible; while peak to peak distances depended on the scan rate; this process was defined as irreversible [40]. The Randles–Sevcik Equation at 298.15 K is:

Ip = (2.99∙10^5^)∙n^3/2^∙A∙C∙D^1/2^∙v^1/2^ (2)

where 2.99 × 10^5^ is a known constant with units C mol^−1^ v^−1/2^, n represents the number of electrons involved in the redox half-reaction (-), D is the diffusion coefficient for the redox-active species in the solution medium (cm^2^ s^−1^), C denotes the solution molar concentration of the redox species (mol cm^−3^), A indicates the surface area (cm^2^), and v represents the scan rate of the experiment (V s^−1^). The diffusion coefficient for 5 mM Ru(NH_3_)_6_Cl_3_ in 0.1 M KCl is 8.43 × 10^−6^ cm^2^ s^−1^, according to Lee et al. [41].

Figures S3 and S4 show the linear regression of the Randles–Sevcik equation for microfeather electrodes. The average active area of the 10 electrodes was 0.7 ± 0.1 cm^2^ which agreed with values reported by different authors [42,43,44]. This value was compared with bare electrodes, proving that the area was highly increased [32].

This parameter correlated to the number of active sites, where glucose oxidation occurs. Increasing the active area is critical for the optimal performance of the sensor. In this sense, the microfeather structure meets all the characteristics required for correct sensor applications.

### 3.3. Linear Range

Cyclic voltammetry was used as the electrochemical technique to determine sensitivity to glucose detection within a wide range of glucose concentrations, from 0 to 20 mM. This range was selected to simulate blood glucose levels. The linear range of microfeathers increased to 8 mM of glucose with a sensitivity of 1091 µA∙Mm^−1^∙cm^−2^. 

Regarding the reaction mechanism, the equations below represent the reaction mechanisms for glucose oxidation with the copper electrode. Equations (6) and (7) occur on the surface of the electrode since its main component is copper oxide, CuO, as discussed previously.

Cu → Cu^2+^ + 2e^−^ (3)

Cu^2+^ + 2OH^−^ → Cu(OH)_2_ (4)

Cu(OH)_2_ → CuO + H_2_O (5)

CuO + OH^−^ → CuOOH + e^−^ (6)

CuOOH + glucose → 2 CuO + glucolactone + H_2_O (7)

**Figure 4:**
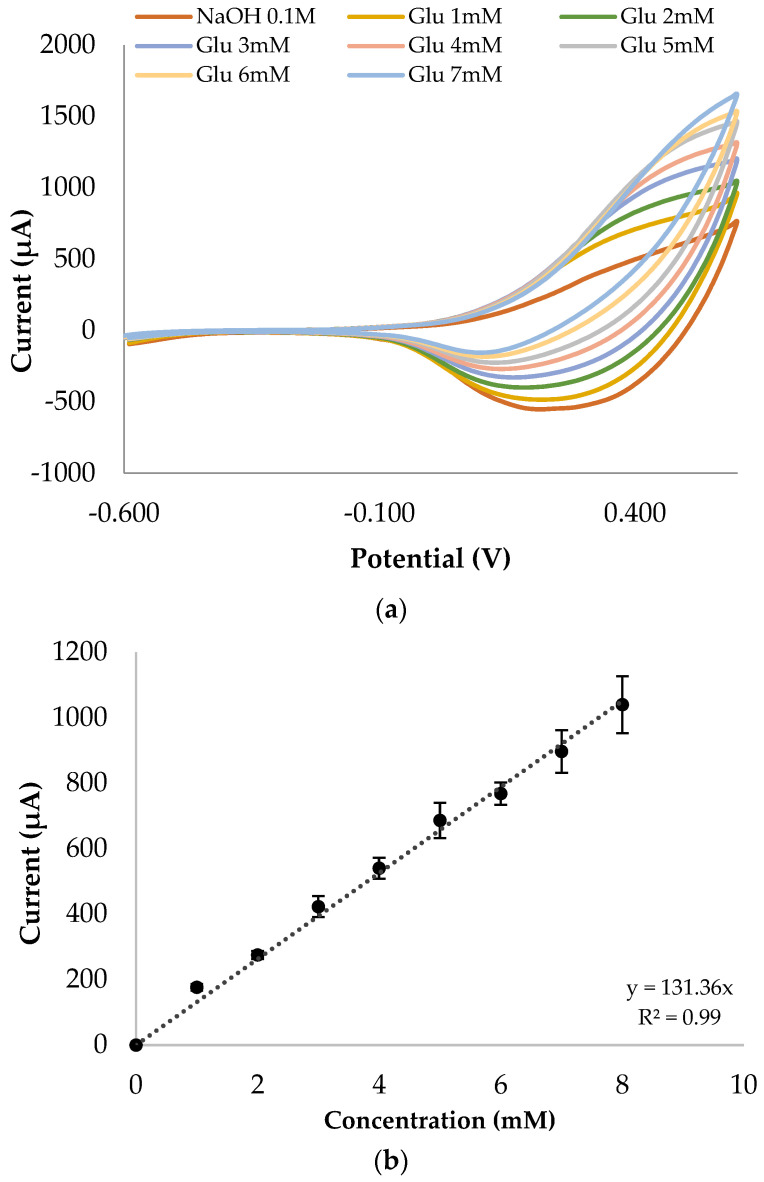
(**a**) Cyclic voltammetry technique (from −0.6 to 0.6 V vs. Ag/AgCl) for glucose detection analysis for 0 to 7 mM in 0.1 M NaOH; (**b**) linear range of the microfeather electrode using cyclic voltammetry technique in 0.1 M NaOH. Values are fixed at +0.55 V vs. Ag/AgCl.

The sensor detection limit was determined at 30 μM. These parameters were established according to the method described by Zare et al. [45]. Figure 4 represents the linear range of the synthetized sensor. 

Three different cyclic voltammetry runs (from −0.6 to 0.6 V vs. Ag/AgCl, 1 cycle) were performed for each concentration, with an error less than 12%. Each electrode was conditioned by developing three 0.1 M NaOH cyclic voltammetry runs.

### 3.4. Reproducibility, Repeatability, and Stability

Repeatability, reproducibility, and stability were studied according to previous protocols. Moreover, actual normative ISO standards were considered since they established actual commercial requirements. Accordingly, the error for glucometers must be less than 15% [46]. 

**Figure 5:**
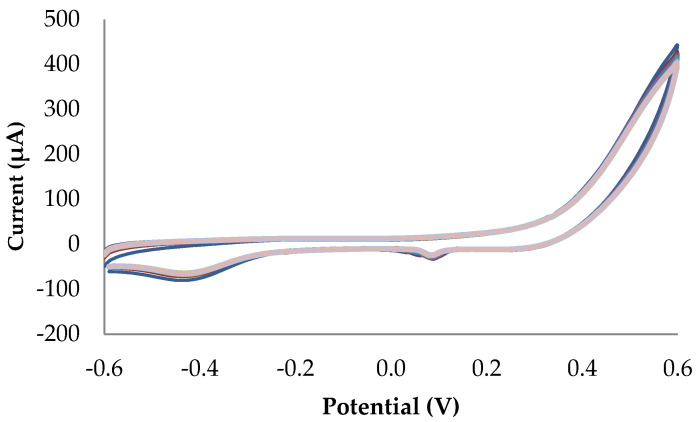
Repeatability of the microfeather electrode; 25 measurements were performed using 3 mM glucose in 0.1 M NaOH.

Figure 5 shows the cyclic voltammetry profile of 25 measurements performed with the same electrode (each color represents a different cyclic voltammetry), generating a repeatability error of 1.9% at +0.55 V vs. Ag/AgCl. This value was very competitive when compared to similar electrodes in the literature. 

**Figure 6:**
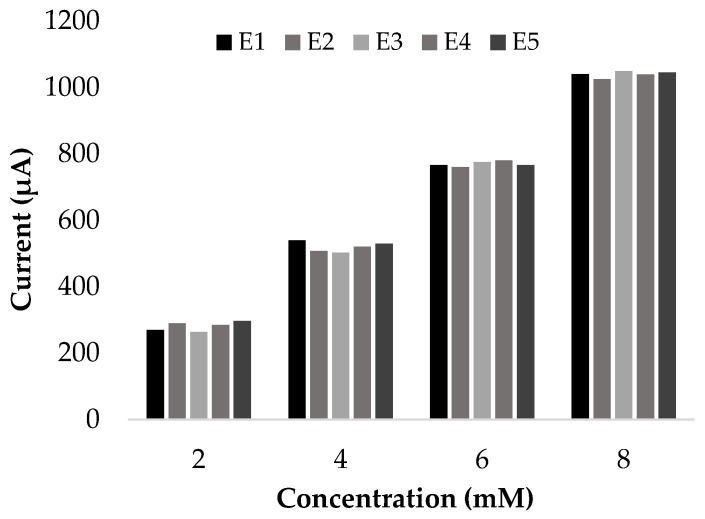
Reproducibility of microfeather electrodes. Measurements with 2, 4, 6, and 8 mM glucose in 0.1 M NaOH (EX where X is the electrode number).

Regarding reproducibility, five different electrodes were tested using different glucose concentrations, as seen in Figure 6. The variation in electrode responses was more significant for high concentrations, generating a maximum error of 7% for the measurement of 4 mmol∙L^−1^. By increasing the concentration to 6 mmol∙L^−1^, this error dropped to 5%. All results met ISO requirements and agreed with results reported by different authors; Fang et al. reported an error of 4% within seven different electrodes that were made with copper Cu(I)/Cu(II) aerogels. Other similar electrodes also reported errors below 5%.

Figure 6 shows the system accuracy according to ISO 15197 standards [46]. For glucose concentrations > 5.55 mmol∙L^−1^, the error must be less than 15%. For concentrations lower than 5.55 mmol∙L^−1^, the allowed deviation was 0.83 mmol∙L^−1^ which translated to an error of 15% at 5.55 mmol∙L^−1^ and 83% at 1 mmol∙L^−1^. This normative was exclusively applied to commercial glucometers designed for biofluids such as blood and interstitial liquid, where glucose concentrations were always above 4 mmol∙L^−1^.

**Figure 7:**
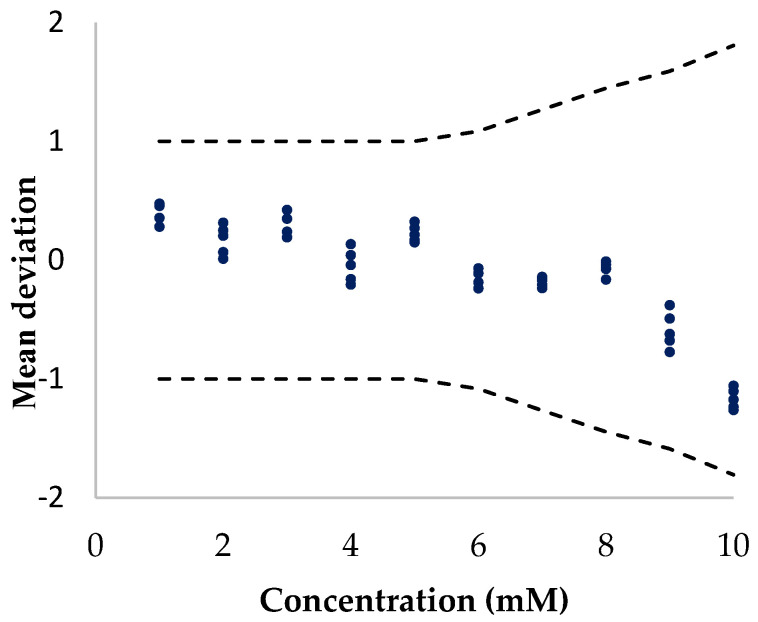
Electrode accuracy according to ISO 15197 standards.

These aspects are shown in Figure 7, where a multiple assay with five different electrodes was carried out to test the validity of microfeather electrodes. Dash line represents the limit provided by ISO 15197 standards.

Again, reproducibility was tested and showed little disparity within electrodes, smaller than 5% RSD (Relative Standard Deviation). Regarding electrode viability for commercial applications, in the case of concentrations lower than 8 mmol∙L^−1^, the system showed a negligible deviation. According to commercial requirements defined by ISO 15197 normative standards, the system was valid up to 10 mmol∙L^−1^. The mean deviation increased with molarity until it surpassed ISO 15197 margins. In this scenario, the copper-based electrode could be proposed for commercial applications since it measured glucose ranges in common biofluids, from 4 mmol∙L^−1^ to 11 mmol∙L^−1^.

Finally, stability and responses under common electrode interferences were studied using Pulsed Amperometry Detection (PAD). This electrochemical method simulates minimally invasive continuous glucometer behavior, where the sensor takes measures every 5 min for almost 2 weeks. In this study, the interval between measurements was reduced to 8 s and measurements were carried out for 2 h every day. Other relevant studies have examined the impact of time over a 3 month period, measuring electrode signals at the beginning and at the end of this period. The results showed a significant decrease in signal strength of 20%. However, this aspect was not evaluated in our work since it did not properly represent the performance of a continuous glucometer. Commercial applications require robust materials to measure in continuous mode in short intervals of time for 2 or more weeks. Testing electrode performance after several months differs from real operations. 

**Figure 8:**
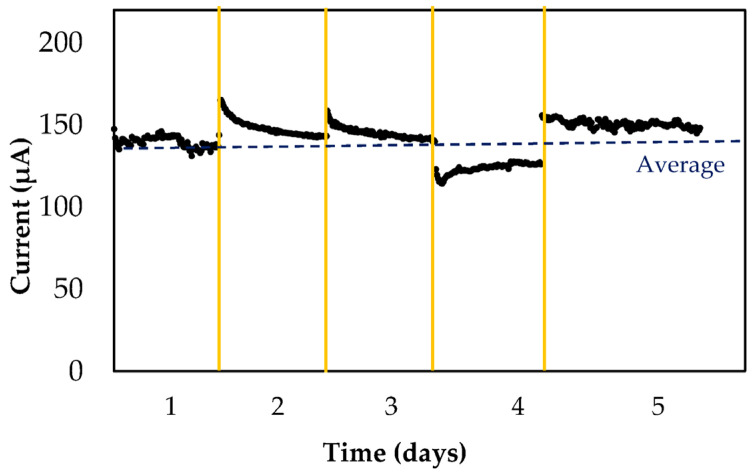
Microfeather electrode stability using PAD at +0.55 V vs. Ag/AgCl. Measurements with 2 mM glucose in 0.1 M NaOH for 5 days. Yellow line represents the separation among days.

Figure 8 shows the stability profile of the microfeather electrode; all measurements were within the range as defined by ISO 15197 standards. The average signal within 5 days was 142 µA with an error of 13% (excluding the daily stabilization measurement). The number of measurements was 4500 while a commercial glucometer only takes 4000 measurements after 2 weeks. In this case, our copper-based electrode maintained its performance for an estimated time of 20 days.

**Figure 9:**
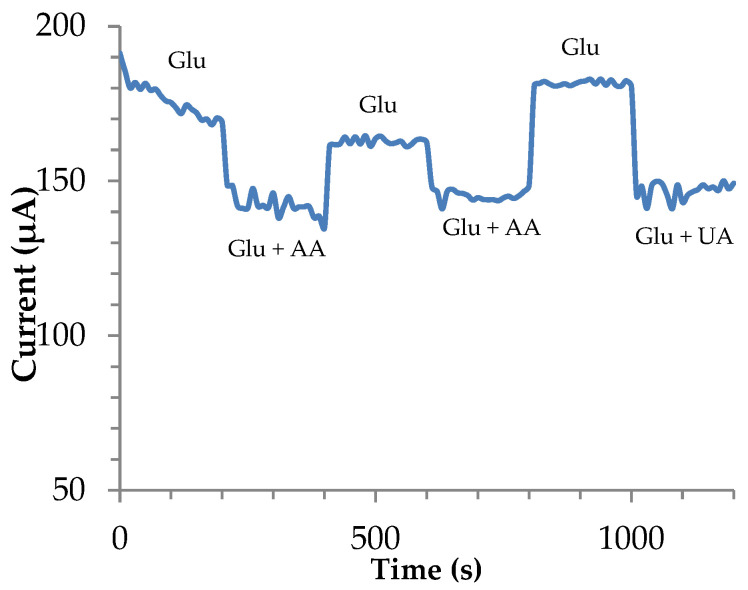
Microfeather electrode interference study using PAD at +0.55 V vs. Ag/AgCl. Measurements with 2 mM glucose in 0.1 M NaOH in the presence of 0.41 mM (UA) and 0.1 mM Ascorbic Acid (AA).

Electrode responses under common interferences are shown in Figure 9. Ascorbic Acid (AA) and Uric acid (UA) were added to 2 mM glucose in 0.1 NaOH samples. Biological ascorbic and uric acid ranges were chosen according to the literature, where ascorbic acid blood levels range from 0.003 to 0.1 mM, while uric acid levels range from 0.15 to 0.45 mM [47,48]. These interferences are also present at lower concentrations in sweat biofluids making this test valid for multiple biofluid applications.

Although the presence of common interferences affected the amperometric signal of the microfeather electrode, the deviation still met commercial requirements. The signal was decreased to only 10% while ISO 15197 standards permitted ± 0.83 mmol∙L^−1^. Finally, our electrode was compared to previous literature results, Table 2 The microfeather electrode provided a good linearity range and good sensitivity compared to other electrodes.

As observed in Table 2, high sensitivity electrodes are specific for small linear range applications, for biofluids with low glucose concentrations, such as lacrimal and urine biofluids. Nowadays, there are no commercial tools for the continuous measurement of glucose in these biofluids; only discrete testing is done for medical reason which does not allow users to control and prevent glycemic alterations such as hyperglycemic peaks after meals. Those sensors with higher linear ranges can be used to detect glucose in interstitial fluid or blood. There is a trade-off between sensitivity and linear detection ranges. For example, CuO/CNT-based electrodes provide a sensitivity of 15,300 μA∙mM^−1^∙cm^−2^ and a linear range which only goes up to 0.1 mM. By contrast, CuO/CS electrodes show a linear range of up to 1 mM with a sensitivity of 503 μA∙mM^−1^∙ cm^−2^. In this scenario, our electrode demonstrated the best options considering sensitivity and linear ranges. The only comparable electrode was CuO/PCA/MWCNT as proposed by Kuznowicz et al. This electrode had a linear range up to 9 mmol∙L^−1^ with a sensitivity of 2412 μA∙mM^−1^∙cm^−2^. However, their catalytic material was formed with more complex substances. In this case, the electrode required poly(Caffeic Acid), multiwalled carbon nanotubes, and copper oxide nanoparticles, while our electrode was made only with a copper precursor and a simple carbon base. This difference significantly impacts on the associated costs of the final product and its viability for macroscale production.

Other studies based on more complex catalyst materials reported very good results for glucose detection in sweat applications; Zha et al. (2022) developed a sensor based on a two-dimensional nanosheet array composed of trimesic acid (H3BTC) and a bimetal metal−organic framework (MOF) on a carbon cloth (CC) [53]. Sweat, as a biofluid for continuous testing, is a potential application since any sensor would be completely non-invasive considering that sweat is accessible without pricking. Several authors have begun to explore this concept using sensor patches, although there are no commercial applications yet [54,55].

## 4. Conclusions

This work reports the optimized synthesis of copper-based microelectrodes for glucose measurements. To this end, a Taguchi experimental design was carried out to ana-lyse the main variables influencing the synthesis process and electrode responses. This methodology provides essential information regarding the key operational variables in developing sensors with minimum costs and time, whilst maintaining outstanding electrode performance. 

Electrode performance was characterized in terms of linear range, repeatability, reproducibility, and stability, with results satisfactorily meeting ISO 15197 commercial requirements and showing robust stability for 5 days. This cost effective and reliable electrode provides high sensitivity of up to 8 mM of glucose, which corresponds with normal blood and interstitial fluid glucose levels. The wide linear range relies on the synthetized micro-structure; microfeathers provide numerous active sites promoting glucose oxidation. The active area increased almost seven times compared to the visual electrode surface which made this structure highly applicable to different sensor industries (healthcare, environment or food industries). In healthcare, this material with its morphology can be combined with other materials to overcome OH dependency, or integrated into a sensor with pH control, which has been widely addressed in the literature. In this scenario, our material will be competitive with commercial sensors that typically last no longer than 2 weeks and involve higher associated costs.

This promising copper-based electrode, synthetized using an inexpensive and simple wet method, offers a robust and affordable catalytic material for the development of new nanostructures and metallic combinations sensitive to physiological conditions. In this sense, non-enzymatic structures will be very competitive with actual glucose sensors based on enzymes. Thus, although further efforts are required in the development of electrodes capable of measuring at neutral pH, this work represents an advance in the field of non-enzymatic sensors for glucose detection in commercial applications. 

## Figures and Tables

**Table 1:** Taguchi parameters used in this work.

Variable	Level 1	Level 2	Level 3
Concentration (I)	0.01 M	0.1 M	1 M
Chrono. Cycles (II)	1	2	3
Chrono. Time (III)	200 s	300 s	400 s
Oxidation time (IV)	2 days	3 days	4 days

**Table 2:** Copper- and copper oxide-based electrodes for glucose sensing.

Electrode	Linear Range (mM)	Reproducibility STD (%)	Sensitivity (μA·mM^−1^·cm^−2^)	Media NaOH	Ref
CuO/CNTs	5–100 μM	1.07	15300	0.1 M	[49]
CuO/rGO/CNT	10–1000 μM	4.1	9278	0.1 M	[42]
CuO/PCA /MWCNT	0.002–9	4.6	2412	0.1 M	[50]
CuO/CS	0.05–1	3.0	503	0.1 M	[51]
Cu_2_O/GCE	0.1–1	N/A	1082.5	0.1 M	[52]
CuO microfeathers	0.03–8	7	1091	0.1 M	This work

PCA = Poly(caffeic acid), CS = chitosan, N/A = Not available

## Data Availability

The data presented in this study are available on request from the corresponding author.

## Supplementary Materials

The following supporting information can be downloaded at: https://www.mdpi.com/article/10.3390/bios13121032/s1. Figure S1. Atomic presence in the microfeather electrode. Figure S2. SEM images; (a) 200× and (b) 2000×. Figure S3. Cyclic voltammetry using 5 mM Ru(NH_3_)_6_Cl_3_ in 0.1M KCl with a scan range from 200 to 20 mV s^−1^. Figure S4. Linear regression of the Randles–Sevcik equation. Table S1. Three Level Orthogonal Array.
