# Supplementary material for: Optimized Copper-Based Microfeathers for Glucose Detection
Source: Biosensors (Basel). 2023 Dec 15;13(12):1032. doi: 10.3390/bios13121032 (PMC10741577; doi:10.3390/bios13121032)
Supplement: Supplementary file 1 [file biosensors-13-01032-s001.zip › biosensors-2735837-supplementary.pdf]

Supplementary Material

Optimized copper based microfeathers for glucose detection

Carlota Guati[1*], Lucía Gomez-Coma[1], Marcos Fallanza[1] and Inmaculada Ortiz[1]

Chemical and Biomolecular Engineering Department, University of Cantabria, 39005 Santander, Spain;

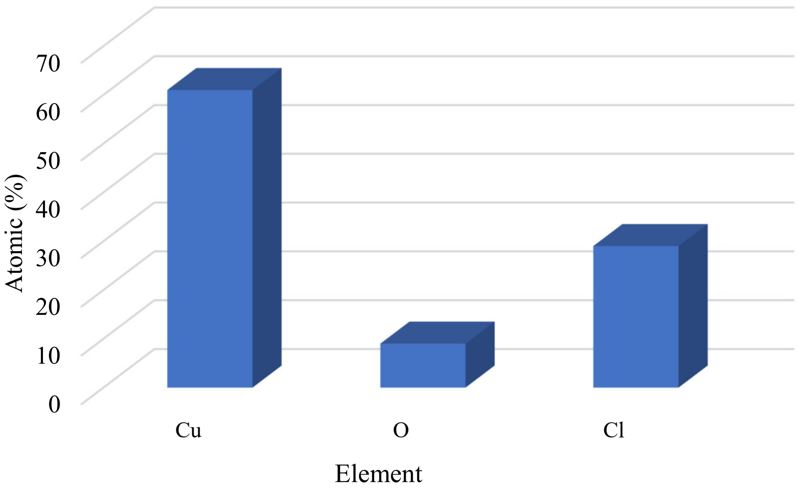

Figure S1. Atomic presence in the microfeather electrode

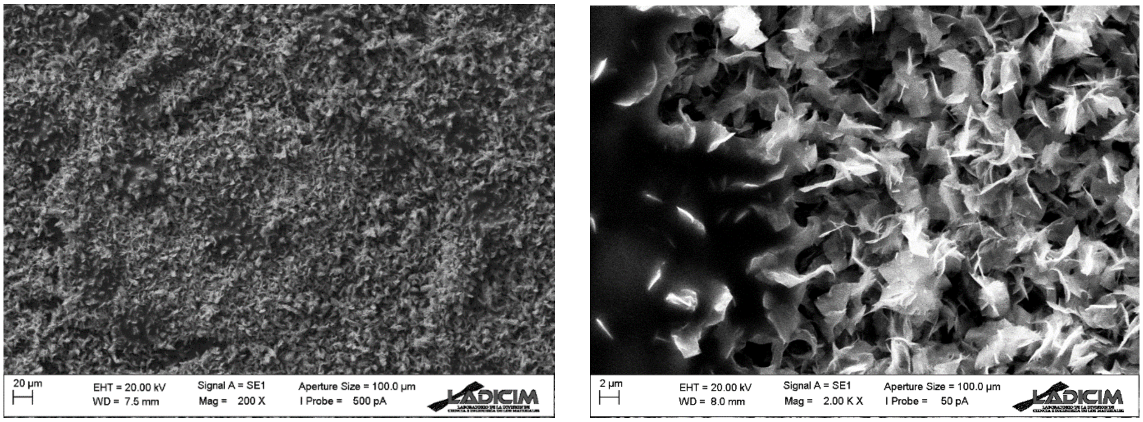

(a) (b)

Figure S2. SEM images; (a) 200× and (b) 2000×

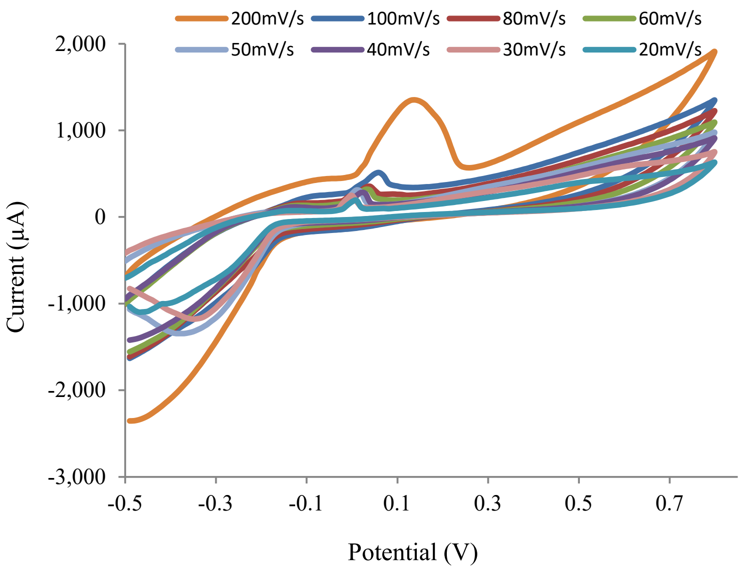

Figure S3. Cyclic voltammetry using 5 mM $Ru(NH_3)_6Cl_3$ in 0.1M KCl with a scan range from 200 to 20 mV s^{-1}

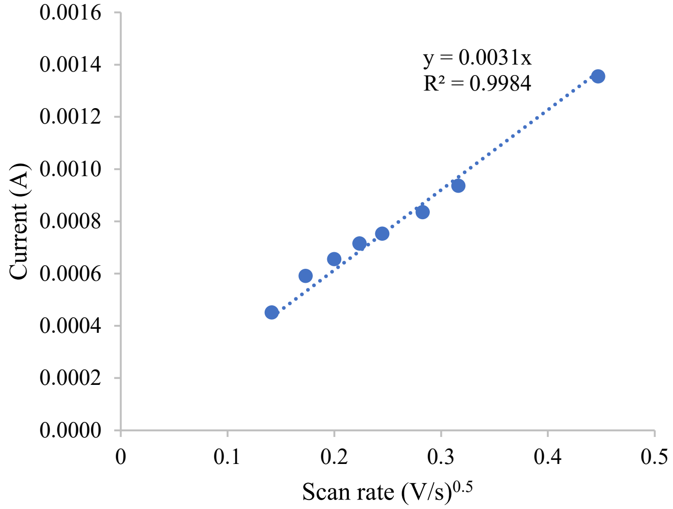

Figure S4. Linear regression of the Randles–Sevcik equation

Table S1. Three Level Orthogonal Array. Ross, P. J. Taguchi Techniques for Quality Engineering. 2nd edn. Edit-ed by McGraw-Hill. 1996

	Column no.			
**Trial no.**	**1**	**2**	**3**	**4**
**1**	1	1	1	1
**2**	1	2	2	2
**3**	1	3	3	3
**4**	2	1	2	3
**5**	2	2	3	1
**6**	2	3	1	2
**7**	3	1	3	2
**8**	3	2	1	3
**9**	3	3	2	1
